# Stimulatory actions of IGF-I are mediated by IGF-IR cross-talk with GPER and DDR1 in mesothelioma and lung cancer cells

**DOI:** 10.18632/oncotarget.10348

**Published:** 2016-06-30

**Authors:** Silvia Avino, Paola De Marco, Francesca Cirillo, Maria Francesca Santolla, Ernestina Marianna De Francesco, Maria Grazia Perri, Damiano Rigiracciolo, Vincenza Dolce, Antonino Belfiore, Marcello Maggiolini, Rosamaria Lappano, Adele Vivacqua

**Affiliations:** ^1^ Department of Pharmacy, Health and Nutritional Sciences, University of Calabria, Rende, Italy; ^2^ Endocrinology, Department of Health Sciences, University Magna Graecia of Catanzaro, Catanzaro, Italy

**Keywords:** DDR1, GPER, IGF-I, IGF-IR, mesothelioma, lung cancer, Pathology Section

## Abstract

Insulin-like growth factor-I (IGF-I)/IGF-I receptor (IGF-IR) system has been largely involved in the pathogenesis and development of various tumors. We have previously demonstrated that IGF-IR cooperates with the G-protein estrogen receptor (GPER) and the collagen receptor discoidin domain 1 (DDR1) that are implicated in cancer progression. Here, we provide novel evidence regarding the molecular mechanisms through which IGF-I/IGF-IR signaling triggers a functional cross-talk with GPER and DDR1 in both mesothelioma and lung cancer cells. In particular, we show that IGF-I activates the transduction network mediated by IGF-IR leading to the up-regulation of GPER and its main target genes CTGF and EGR1 as well as the induction of DDR1 target genes like MATN-2, FBN-1, NOTCH 1 and HES-1. Of note, certain DDR1-mediated effects upon IGF-I stimulation required both IGF-IR and GPER as determined knocking-down the expression of these receptors. The aforementioned findings were nicely recapitulated in important biological outcomes like IGF-I promoted chemotaxis and migration of both mesothelioma and lung cancer cells. Overall, our data suggest that IGF-I/IGF-IR system triggers stimulatory actions through both GPER and DDR1 in aggressive tumors as mesothelioma and lung tumors. Hence, this novel signaling pathway may represent a further target in setting innovative anticancer strategies.

## INTRODUCTION

Lung cancer is the most frequent cause of cancer incidence and mortality worldwide at least in part due to the increasing number of risk factors in diverse developing countries [1-2]. To date, smoking has been considered the main etiologic factor for lung cancer [3-4], however, several environmental contaminants like asbestos, arsenic, cadmium, nickel and silica, play an important role toward the development of this neoplasia [5]. Among the aforementioned environmental pollutants, asbestos has been particularly acknowledged as prompting factor in malignant mesothelioma (MM), which is an aggressive cancer that arises from mesothelial cells lining lung, pleura or peritoneum [6-7]. Chronic inflammatory processes caused by the deposition of asbestos fibers and the subsequent release of cytokines and growth factors by macrophages and mesothelial cells have been shown to play an active role toward the development of both pleural MM and lung cancer [7-8].

In this vein, the IGF system, the complex system involving the insulin-like growth factors (IGFs) and related receptors as well as IGF-binding proteins, has been established as an important regulator of tumor initiation and progression in several malignancies, including pleural MM and lung cancer [9-13]. In particular, the IGF-I receptor (IGF-IR), which is often overexpressed in diverse cancer cell types, affects tumor development, progression and resistance to therapies [11, 14-16]. Moreover, a dysregulated IGF system has been shown to be implicated in various chronic diseases, such as pulmonary fibrosis [17-18].

An increasing body of data has demonstrated that the biological responses mediated by IGF-I involve functional interactions of IGF-IR with diverse signal molecules belonging to other members of the receptor tyrosine kinase (RTK) family [19-20]. In this context, we recently discovered a novel functional cross-talk between IGF-IR and the collagen receptor discoidin domain receptor 1 (DDR1), a molecule also overexpressed in diverse malignancies, including lung carcinomas, and implicated in cancer progression [21]. Interestingly, this cross-talk occurs also independently of the collagen binding actions of DDR1 and, in human breast cancer cells, amplifies the stimulatory biological effects of IGF-I toward proliferation, migration and colony formation. Moreover, through a signaling pathway involving Akt/miR-199a-5p, IGF-I is able to upregulate DDR1 [12, 22].

In addition to RTKs, IGF-IR interacts with other important signaling molecules like G protein-coupled receptors (GPCRs) [19, 23]. These functional interactions have also important implications in the development and progression of diverse types of tumors [23-24]. In particular, we found that IGF-IR activation engages the G protein estrogen receptor (GPER/GPR30)-mediated signaling toward the stimulation of proliferation and migration of different cancer cell types [25-26]. Interestingly, high expression levels of GPER were detected in lung cancer cells and involved in growth stimulatory effects [24, 27-28]. To date, other signaling molecules have been implicated in the development of MM including the estrogen receptor (ER)β that may act as a tumor suppressor [29-30]. Therefore, the multifaceted mechanisms and the transduction network of factors involved in the progression of the aforementioned malignancies remain to be fully understood.

In this study, we found that mesothelioma and lung cancer cells show a new complex functional cross-talk involving IGF-IR, GPER and DDR1, which affects gene expression and biological effects in response to IGF-I. Our data, therefore, further extend the molecular mechanisms by which IGF-I may affect tumor progression in mesothelioma and lung cancer, hence providing novel targets in the aforementioned aggressive malignancies.

## RESULTS

### IGF-I stimulates GPER expression through IGF-IR/ERK/p-38 transduction signaling

**Figure 1:**
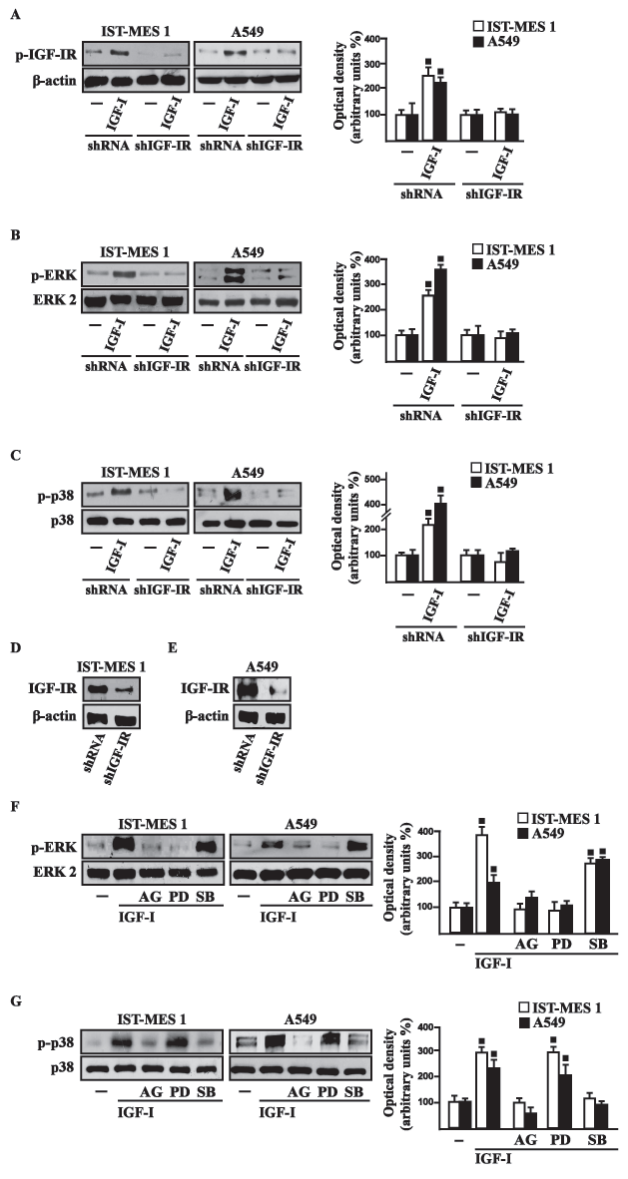
Rapid activation of transduction signaling by IGF-I in IST-MES 1 and A549 cells IGF-IR **A.**, ERK **B.** and p-38 **C.** phosphorylation in cells transfected for 24 h with shRNA or shIGF-IR treated with vehicle (-) or 100 ng/ml IGF-I for 15 min. **D.-E.** Efficacy of IGF-IR silencing. ERK **F.** and p-38 **G.** activation in cells treated for 15 min with vehicle (-) or 100 ng/ml IGF-I alone and in combination with either 1 μM IGF-IR inhibitor tyrphostin AG1024 (AG), or 1 μM MEK inhibitor PD98059 (PD) or 1 μM p38 inhibitor SB202190 (SB). Side panels show densitometric analysis of the blots normalized to β-actin, ERK2 and p38 that served as loading controls respectively for pIGF-IR, pERK and p-p38. Data shown are the mean ± SD of three independent experiments. (◾) *p* < 0.05 for cells receiving vehicle (-) *versus* treatments.

**Figure 2:**
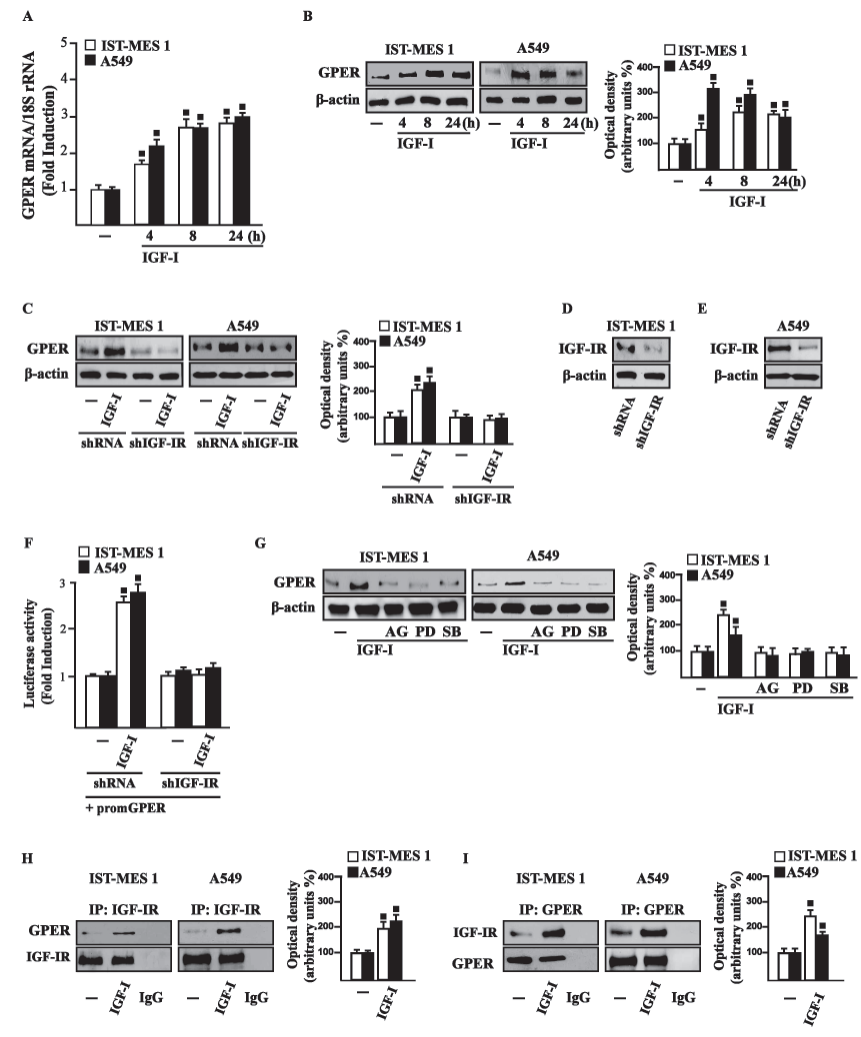
IGF-I up-regulates GPER expression in IST-MES 1 and A549 cells **A.** mRNA expression of GPER in cells treated with either vehicle (-) or 100 ng/ml IGF-I, as evaluated by real-time PCR. Results obtained from experiments performed in triplicate were normalized for 18S expression and shown as fold change of RNA expression compared to cells treated with vehicle. **B.** GPER protein levels were evaluated by immunoblotting in cells treated with either vehicle (-) or 100 ng/ml IGF-I, as indicated. **C.** GPER protein expression in cells transfected for 24 h with either shRNA or shIGF-IR and then treated for 8 h with vehicle (-) or 100 ng/ml IGF-I. **D.-E.** Efficacy of IGF-IR silencing. **F.** Cells were transfected for 24 h with shRNA or shIGF-IR together with the GPER promoter construct. Then, cells were treated for 18 h with vehicle (-) or 100 ng/ml IGF-I. The luciferase activities were normalized to the internal transfection control, and values of cells receiving vehicle (-) were set as one fold induction upon which the activity induced by treatments was calculated. **G.** GPER protein levels in cells treated for 8 h with vehicle (-) or 100 ng/ml IGF-I alone or in combination with 1 μM IGF-IR inhibitor tyrphostin AG1024 (AG), 1 μM MEK inhibitor PD98059 (PD) and 1 μM p38 inhibitor SB202190 (SB). Side panels show densitometric analysis of the blots normalized to β-actin. **H.-I.** Co-immunoprecipitation studies performed in cells treated for 8 h with vehicle (-) or 100 ng/ml IGF-I, as indicated. In control samples, non-specific IgG was used instead of the primary antibody. **H.** Side panel show densitometric analysis of the blot normalized to IGF-IR. **I.** Side panel show densitometric analysis of the blot normalized to GPER. Data shown are the mean ± SD of three independent experiments. (◾) *p* < 0.05 for cells receiving vehicle (-) *versus* treatments.

On the basis of previous studies showing that IGF-I triggers stimulatory effects in malignant mesothelioma as well as in lung cancer cells [31-32], we began our study evaluating the transduction signaling activated by IGF-I in IST-MES1 mesothelioma and A549 lung cancer cells, which were used as model system. First, we determined that in both cell types IGF-I induces the phosphorylation of IGF-IR (Figure 1A) and both ERK (Figure 1B) and p-38 (Figure 1C). As expected, these responses were no longer observed after IGF-IR silencing (Figure 1A-1E). The activation of ERK triggered by IGF-I was abolished in the presence of the IGF-IR inhibitor AG and the MEK inhibitor PD, but it still persisted using the p-38 inhibitor SB (Figure 1F). The phosphorylation of p-38 was prevented by AG and SB, but not in the presence of PD (Figure 1G). In addition, we assessed that the phosphorylation of IGF-IR induced by IGF-I is inhibited exclusively by AG, but not in the presence of PD and SB (data not shown), then suggesting that the activation of both ERK and p-38 relies directly on IGF-IR phosphorylation upon IGF-I exposure. On the basis of our previous data showing that IGF-I signaling cooperates with several GPCR family members, including GPER, toward cancer progression [19, 25], we evaluated whether IGF-I regulates GPER expression in IST-MES1 and A549 cells. In this regard, time-course experiments demonstrated that IGF-I up-regulates GPER at both mRNA (Figure 2A) and protein levels (Figure 2B). Moreover, we ascertained that these responses to IGF-I occurred through IGF-IR, as the induction of GPER mRNA (data not shown) and protein levels (Figure 2C-2E) was abolished by knocking-down IGF-IR expression. Recapitulating the aforementioned findings, the transactivation of the GPER promoter by IGF-I was prevented by IGF-IR silencing (Figure 2F), and the IGF-I induced GPER protein up-regulation was abrogated in the presence of AG, PD and SB (Figure 2G). Taken together, these results indicate that the IGF-I/IGF-IR transduction pathway stimulates GPER expression through ERK and p-38 signaling. In order to further investigate this functional cross-talk between IGF-IR and GPER, we performed co-immunoprecipitation studies determining that IGF-I triggers also a direct interaction between these receptors in both IST-MES1 and A549 cells upon either 1 h (data not shown) or 8 h treatment with IGF-I (Figure 2H-2I), thus suggesting that the interaction between IGF-IR and GPER may occur without a newly protein expression of GPER.

### IGF-I triggers the expression of GPER target genes

**Figure 3:**
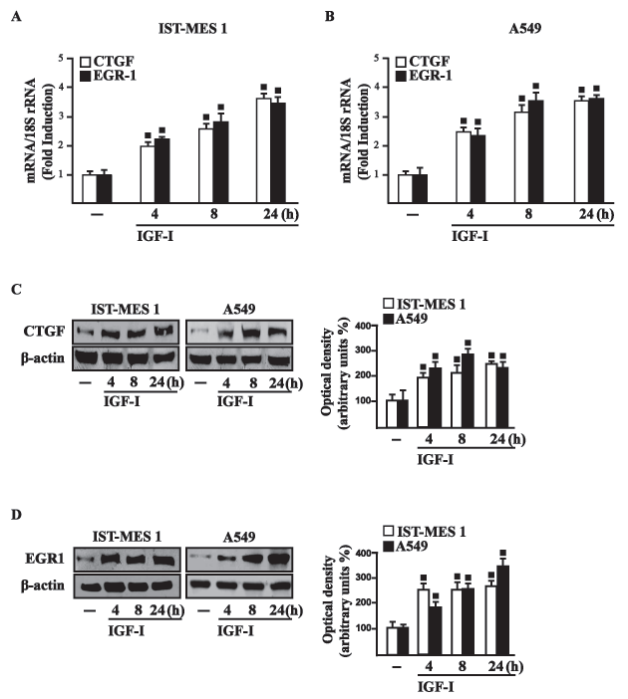
IGF-I up-regulates CTGF and EGR1 expression in IST-MES 1 and A549 cells (A-B) mRNA expression of CTGF and EGR1 in cells treated with either vehicle (-) or 100 ng/ml IGF-I, as evaluated by real-time PCR. Results obtained from experiments performed in triplicate were normalized for 18S expression and shown as fold change of RNA expression compared to cells treated with vehicle. CTGF **C.** and EGR1 **D.** protein levels were evaluated by immunoblotting in cells treated with vehicle (-) or 100 ng/ml IGF-I, as indicated. Side panels show densitometric analysis of the blots normalized to β-actin and each data point represents the mean ± SD of three independent experiments. (◾) *p* < 0.05 for cells receiving vehicle (-) *versus* treatments.

**Figure 4:**
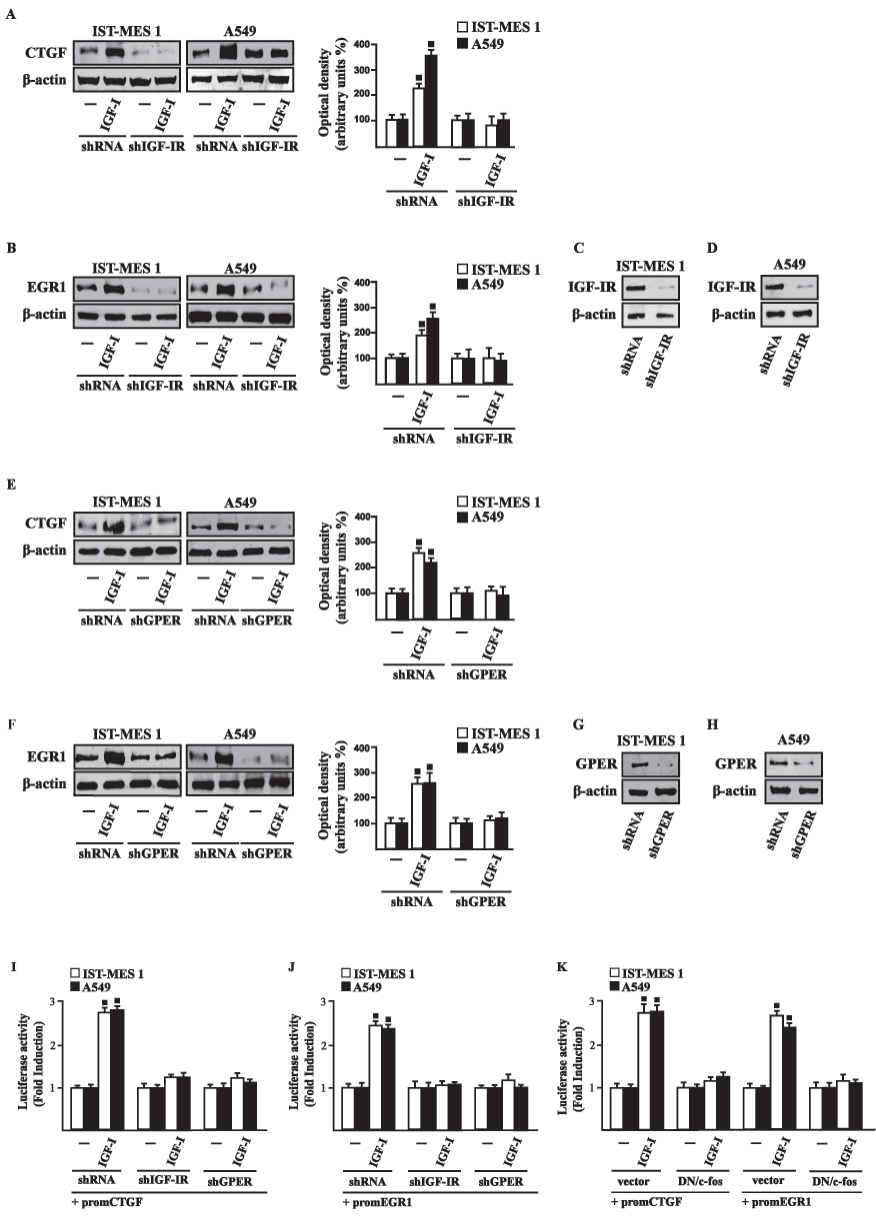
IGF-IR and GPER mediate CTGF and EGR1 stimulation by IGF-I in IST-MES 1 and A549 cells **A.-F.** CTGF and EGR1 protein levels in cells transfected for 24 h with shRNA, shIGF-IR or shGPER and then treated for 8 h with either vehicle (-) or 100 ng/ml IGF-I. Efficacy of IGF-IR **C.-D.** and GPER **G.-H.** silencing. Side panels show densitometric analysis of the blots normalized to β-actin. **I.-J.** Cells were transfected for 24 h with shRNA, shIGF-IR or shGPER together with the CTGF or EGR1 promoter construct. Then, cells were treated for 18 h with vehicle (-) or 100 ng/ml IGF-I. **K.** Cells were transfected for 24 h with a dominant negative form of c-fos (DN/c-fos) together with the CTGF or EGR1 promoter construct. Then, cells were treated for 18 h with vehicle (-) or 100 ng/ml IGF-I. The luciferase activities were normalized to the internal transfection control, and values of cells receiving vehicle (-) were set as one fold induction upon which the activity induced by treatments was calculated. Data shown are the mean ± SD of three independent experiments. (◾) *p* < 0.05 for cells receiving vehicle (-) *versus* treatments.

In our previous study [33] we established that GPER mediates a specific gene signature, therefore, we evaluated whether, in IST-MES1 and A549 cells, IGF-I is able to affect the expression of certain GPER target genes like CTGF and EGR1, which have been involved in fibrotic responses in mesothelioma and lung cancer cells [34-36]. Indeed, in time-course experiments we found that IGF-I increases the mRNA (Figure 3A-3B) and protein levels (Figure 3C-3D) of both CTGF and EGR1. Next, we determined that this action of IGF-I involves not only the IGF-IR but also GPER, as the silencing of each of these receptors prevented gene changes (Figure 4A-4H). In accordance with these observations, the IGF-I transactivation of CTGF (Figure 4I) and EGR1 (Figure 4J) promoters required both IGF-IR and GPER, as demonstrated by knocking down the expression of these receptors. As c-fos plays a main role in the up-regulation of GPER target genes [33, 37], we next determined that the promoter transactivation of both CTGF and EGR1 is abrogated by co-transfecting a dominant-negative form of c-fos (DN/c-fos) in IST-MES1 and A549 cells (Figure 4K). Collectively, these findings provide novel mechanisms through which IGF-I/IGF-IR transduction signaling regulates GPER target genes like CTGF and EGR1 in mesothelioma and lung cancer cells.

### IGF-IR and GPER are both involved in IGF-I regulation of DDR1 target genes

**Figure 5:**
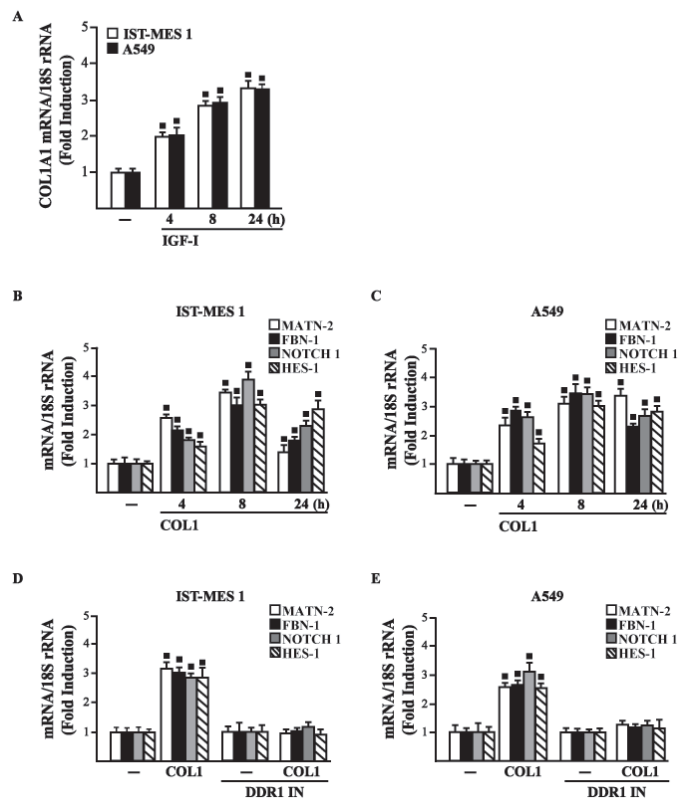
**A.** mRNA expression of COL1A1 in IST-MES 1 and A549 cells treated with vehicle (-) or 100 ng/ml IGF-I, as evaluated by real-time PCR. mRNA expression of MATN-2, FBN-1, NOTCH 1 and HES-1 in IST-MES 1 **B.**, **D.** and A549 **C.**, **E.** cells treated with vehicle (-) or 10 μg/ml COL1 alone or in combination with 1 μM DDR1 inhibitor (DDR1 IN), as indicated. Results obtained from experiments performed in triplicate were normalized for 18S expression and shown as fold change of RNA expression compared to cells treated with vehicle. (◾) *p* < 0.05 for cells receiving vehicle (-) *versus* treatments.

**Figure 6:**
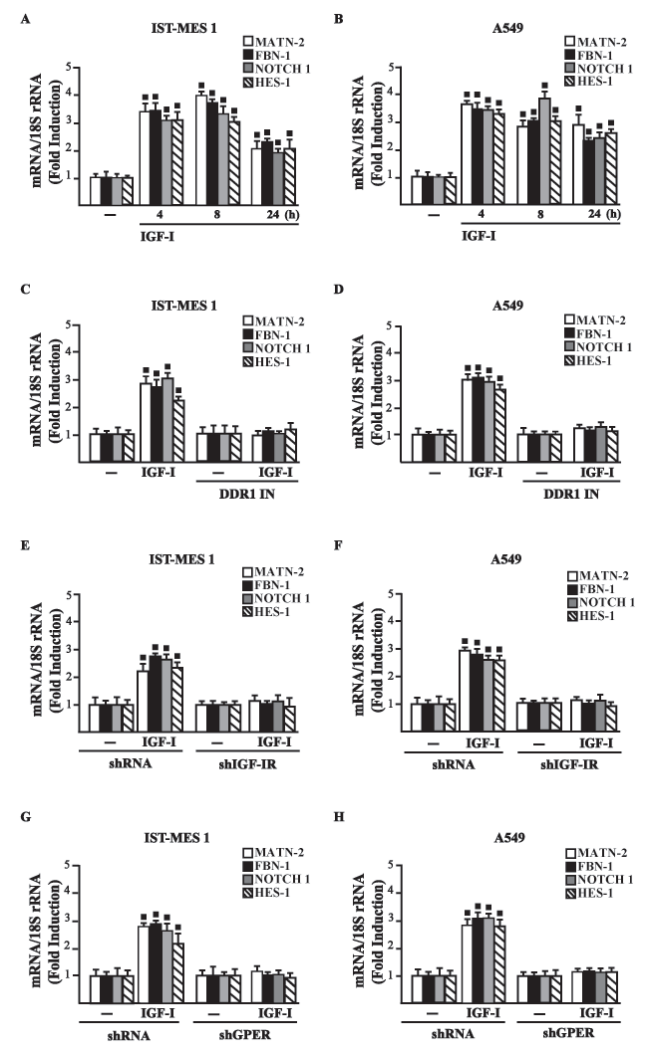
IGF-IR and GPER mediate the IGF-I induced up-regulation of COL1A1/DDR1 target genes in IST-MES 1 and A549 cells **A.-D.** mRNA expression of MATN-2, FBN-1, NOTCH 1 and HES-1 in cells treated with vehicle (-) or 100 ng/ml IGF-I alone or in combination with 1 μM DDR1 inhibitor (DDR1 IN), as indicated. **E.-H.** mRNA expression of MATN-2, FBN-1, NOTCH 1 and HES-1 in cells transfected for 24 h with shRNA, shIGF-IR or shGPER and then treated for 8 h with vehicle (-) or 100 ng/ml IGF-I. Results obtained from experiments performed in triplicate were normalized for 18S expression and shown as fold change of RNA expression compared to cells treated with vehicle. (◾) *p* < 0.05 for cells receiving vehicle (-) *versus* treatments.

**Figure 7:**
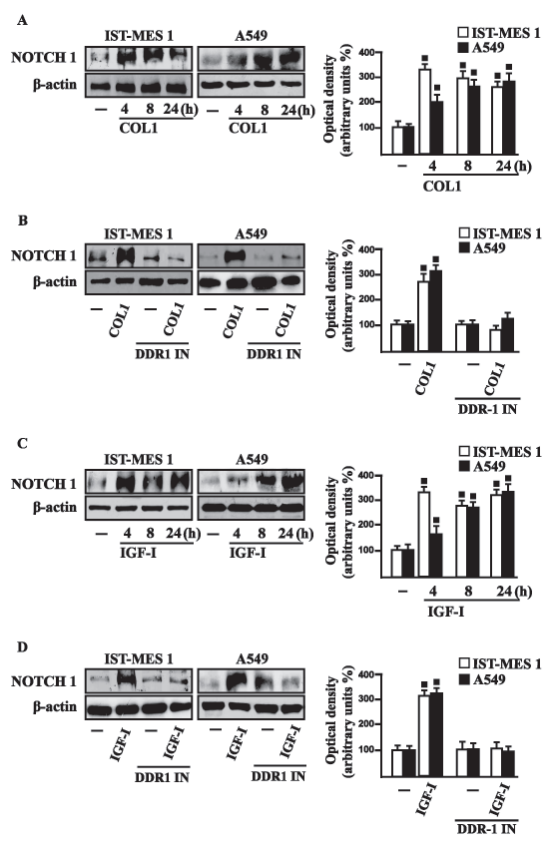
COL1 and IGF-I stimulate NOTCH 1 expression through DDR1 in IST-MES 1 and A549 cells **A.** NOTCH 1 protein levels in cells treated with vehicle (-) or 10 μg/ml COL1, as indicated. **B.** NOTCH 1 protein levels in cells treated for 8 h with vehicle (-) or 10 μg/ml COL1 alone and in combination with 1 μM DDR1 inhibitor (DDR1 IN). **C.** NOTCH 1 protein levels in cells treated with vehicle (-) or 100 ng/ml IGF-I, as indicated. **D.** NOTCH 1 protein levels in cells treated for 8 h with vehicle (-) or 100 ng/ml IGF-I alone and in combination with 1 μM DDR1 inhibitor (DDR1 IN). Side panels show densitometric analysis of the blots normalized to β-actin and each data point represents the mean ± SD of three independent experiments. (◾) *p* < 0.05 for cells receiving vehicle (-) *versus* treatments.

**Figure 8:**
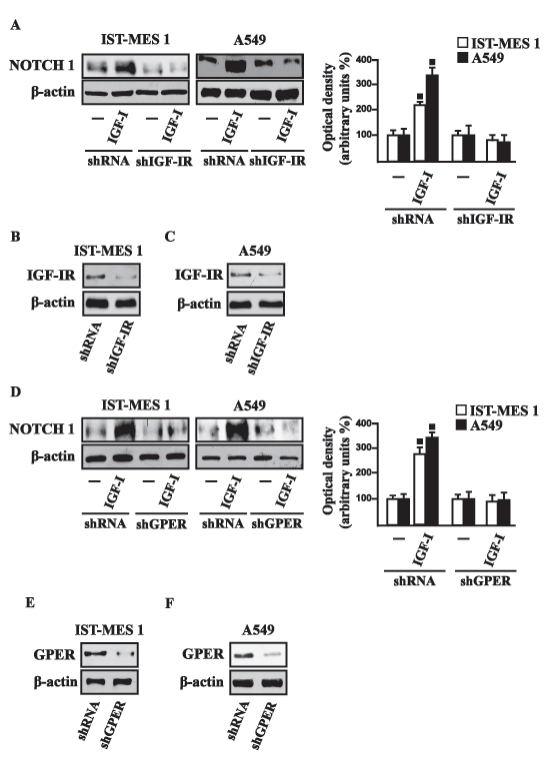
IGF-IR and GPER mediate the IGF-I induced up-regulation of NOTCH 1 in IST-MES 1 and A549 cells NOTCH 1 protein levels in cells transfected for 24 h with shIGF-IR **A.** or shGPER **D.** and then treated for 8 h with vehicle (-) or 100 ng/ml IGF-I. Efficacy of IGF-IR **B.-C.** and GPER **E.-F.** silencing. Side panels show densitometric analysis of the blots normalized to β-actin. (◾) *p* < 0.05 for cells receiving vehicle (-) *versus* treatments.

Considering that in diverse model systems IGF-I stimulates the synthesis of collagen [38-40], we next established that IGF-I regulates in both IST-MES1 and A549 cells the mRNA expression of COL1A1 (Figure 5A) that encodes the major component of type I collagen [41]. We previously reported that IGF-IR functionally interacts with DDR1, which is activated by various collagen types including type I collagen. Therefore, we first ascertained that, in both IST-MES1 and A549 cells, several DDR1 target genes such as matrilin-2 (MATN-2), fibrillin-1 (FBN-1), NOTCH 1 and HES-1, are induced by the DDR1 agonist COL1 (Figure 5B-5C) and abrogated by the DDR1 inhibitor (DDR1 IN) (Figure 5D-5E). Then, we assessed that these DDR1 target genes are also stimulated by IGF-I (Figure 6A-6B) and that this response was inhibited by DDR1 IN (Figure 6C-6D) as well as by silencing IGF-IR (Figure 6E-6F) or GPER (Figure 6G-6H). In accordance with these findings, we determined that the NOTCH 1 protein induction by COL1 and IGF-I is prevented in the presence of the DDR1 IN in IST-MES1 and A549 cells (Figure 7). Accordingly, IGF-I was not able to trigger NOTCH 1 protein expression when IGF-IR (Figure 8A-8C) or GPER (Figure 8D-8F) were silenced. Altogether, these results indicate that, in both mesothelioma and lung cancer cells, IGF-I may up-regulate DDR1 target genes, and this action involves not only IGF-IR but also a cross-talk with GPER.

### DDR1, IGF-IR and GPER contribute to the chemotaxis and migration of mesothelioma and lung cancer cells

**Figure 9:**
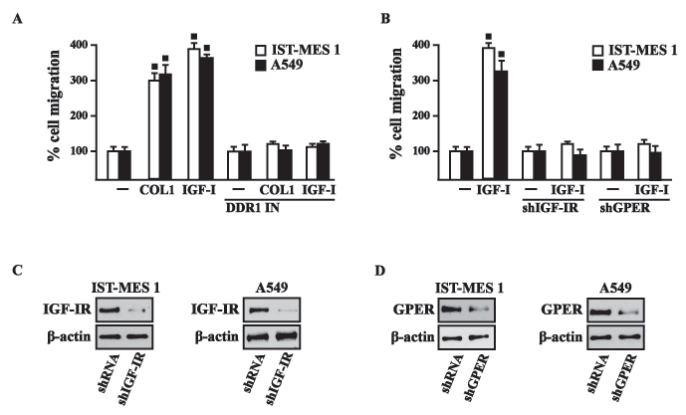
COL1 and IGF-I stimulate IST-MES 1 and A549 cell migration through DDR1, IGF-IR and GPER **A.** The migration of IST-MES 1 and A549 cells upon 8 h treatment with vehicle (-), 10 μg/ml COL1 or 100 ng/ml IGF-I alone and in combination with 1 μM DDR1 inhibitor (DDR1 IN), as evaluated by Boyden Chamber assay. **B.** The migration of IST-MES 1 and A549 cells induced by 8 h treatment with 100 ng/ml IGF-I was prevented knocking down IGF-IR and GPER expression, as evaluated by Boyden Chamber assay. Efficacy of IGF-IR **C.-D.** and GPER **E.-F.** silencing. Values represent the mean ± SD of three independent experiments. (⦁) indicates *p* < 0.05 for cells treated with vehicle (-) *versus* treatments.

Previous studies have reported that IGF-I stimulates chemotactic and chemokinetic motility in mesothelioma cells [32]. Moreover, DDR1 also plays an important role in promoting cell-cell interactions and cell migration in various cell contexts [42-45]. Further extending these data, in IST-MES1 cells, we found that both IGF-I and COL1 induce chemotactic motility, which requires DDR1, as these responses were abolished by DDR1 IN (Videos 1-6). Moreover, we ascertained that the chemotactic motility induced by IGF-I requires also IGF-IR and GPER as the aforementioned effect was prevented silencing the expression of these receptors (Videos 7-12). Similar findings occurred in A549 cells (data not shown). Likewise, we determined that IST-MES1 and A549 cell migration induced by both IGF-I and COL1 is abolished using DDR1 IN (Figure 9A), whereas the silencing of IGF-IR or GPER abolished cell migration triggered by IGF-I, as determined by Boyden chamber assay (Figure 9B). Collectively, our data indicate novel cross-talk and biological functions exerted by IGF-I toward tumor progression.

## DISCUSSION

**Figure 10:**
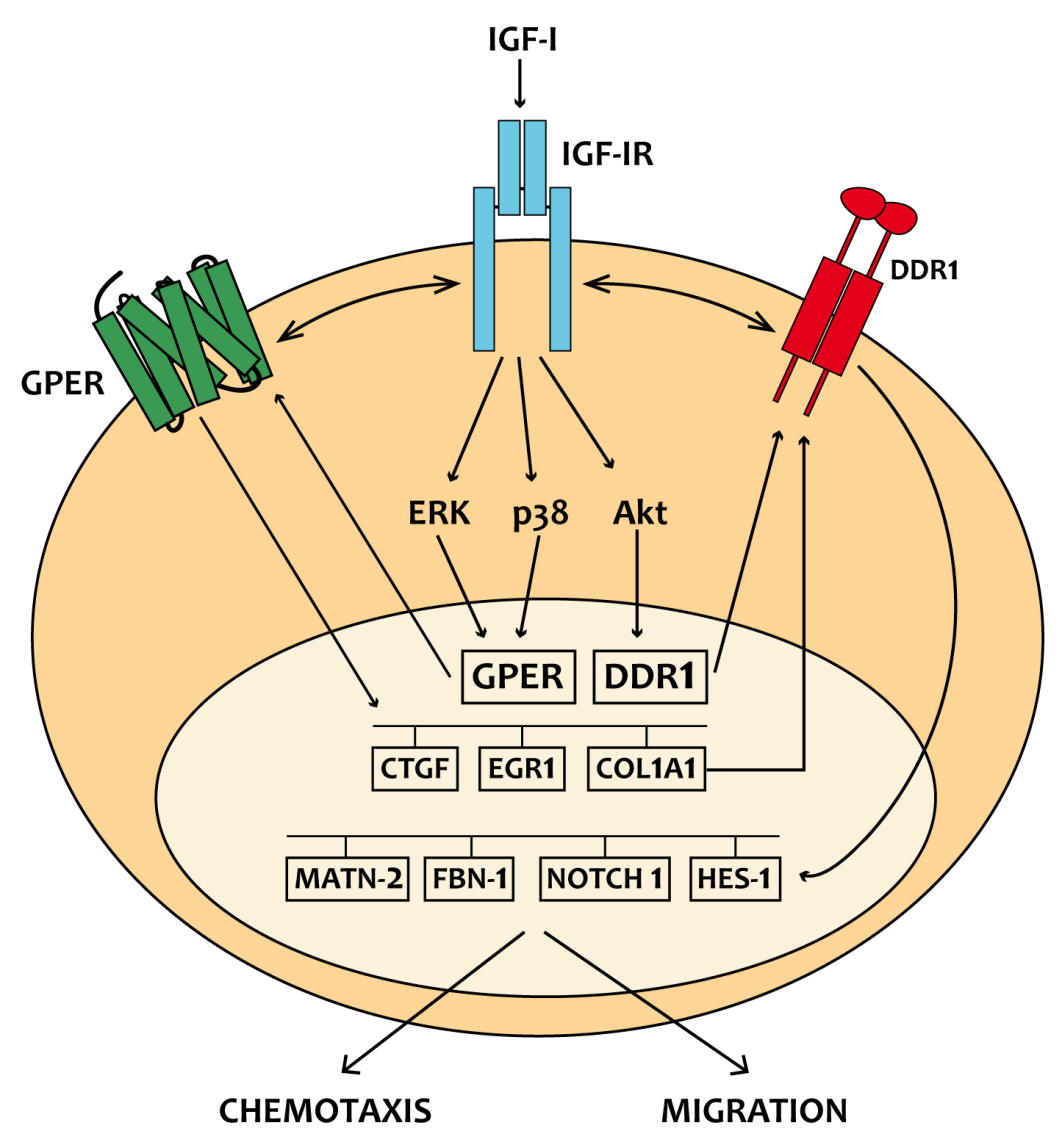
Schematic representation of the signaling network between IGF-IR, GPER and DDR1 activated by IGF-I IGF-I stimulates the expression of GPER and its target genes, then IGF-IR and GPER trigger the IGF-I regulation of DDR1 target genes. The functional cross-talk of IGF-IR, GPER and DDR1 contributes to the chemotaxis and migration observed in cancer cells.

In the present study we provide novel evidence regarding the molecular mechanisms by which IGF-I triggers biological responses in mesothelioma and lung cancer cells. In particular, we show a complex functional cooperation involving IGF-IR, GPER and DDR1 through which IGF-I up-regulates first the expression of COL1A1 and certain DDR1 target genes, thereafter stimulating cancer cell motility and chemotactic response (Figure 10).

Lung cancer is a highly heterogeneous tumor that can arise in different sites of the bronchial tree [1-2]. The incidence of lung cancer depends on toxic effects of inhaled substances such as tobacco, asbestos, arsenic, cadmium, nickel and silica [46]. The environmental pollutant asbestos is also considered the main cause of the insurgence of malignant mesothelioma (MM), which is a rare and aggressive tumor that springs from mesothelial cells lining lung, pleura or peritoneum [5-7, 47-48]. The deposition of asbestos fibers has been also related to chronic inflammatory processes as well as to pulmonary fibrosis, which in turn may create a favorable environment for the development of lung and pleura malignancies [6, 49]. As it concerns the multifaceted mechanisms and factors involved in pulmonary fibrosis and neoplasia, an increased expression and activation of DDR1 have been reported [50-53]. To date, DDR1 has been shown to play an important role in cancer progression by regulating the interactions of tumor cells with the surrounding collagen matrix, therefore leading to pro-migratory and pro-invasive responses [21]. Furthermore, collagen activated DDR1 triggers diverse pro-survival pathways toward anti-apoptotic, proliferative and aggressive features in cancer cells [21]. In this regard, it should be noted that several types of collagen are able to bind to and activate DDR1, which then regulates cell and tissue homeostasis acting as a collagen sensor [21, 54]. Of note, an abnormal expression and deposition of collagen has been associated with cancer development [55-56]. As it concerns the synthesis and extracellular accumulation of diverse types of collagen, cytokines and growth factors like IGF-I, the epidermal growth factor (EGF) and the transforming growth factor-βl have been reported to promote these effects [38-40, 57]. Notably, we previously showed that, in breast cancer cells, IGF-I may upregulate DDR1 expression through a signaling pathway involving the DDR1 regulatory miR-199a-5p [12]. Moreover, the activation of one of the main IGF-I transduction signaling, the IGF-IR/PI3K/Akt cascade, inhibits miR-199a-5p expression, thus relieving its inhibition upon DDR1 gene and allowing DDR1 upregulation. In turn, DDR1 increases IGF-IR expression through post-transcriptional mechanisms and amplifies IGF-I downstream signaling and biological effects, such as proliferation, migration and colony formation [12]. Indeed, we previously showed that DDR1 directly interacts with IGF-IR, and that this interaction is enhanced by IGF-I stimulation, which promotes rapid DDR1 tyrosine-phosphorylation and co-internalization of the DDR1 - IGF-IR complex [22]. This interaction was shown to occur in a panel of human breast cancer cells as well as in mouse fibroblasts (R- cells) co-transfected with the human IGF-IR and DDR1, indicating that it is not cell-specific. Notably, the formation of this DDR1 - IGF-IR complex did not require the presence of collagen, the canonical DDR1 ligand. In addition, the critical role of IGF-IR in DDR1 activation and biological actions is supported by the finding that collagen-dependent DDR1 phosphorylation was impaired in the absence of IGF-IR [22].

Extending these previous studies, we now show that IGF-I through the cognate receptor IGF-IR is able to induce COL1A1 expression [54]. Moreover, a panel of DDR1 target genes could be also induced by IGF-I through the previously described functional cross-talk involving IGF-IR and DDR1. Taken together, these findings show that DDR1, besides enhancing the activation of typical IGF-IR downstream cascades, the PI3K/Akt and the ERK1/2 cascades, following cell exposure to IGF-I, modifies significantly these IGF-I effects by allowing the induction of typical DDR1 target genes. These effects confirm the relevance of DDR1 in the amplification and diversification of IGF-I signaling pathways in cancer. We have previously demonstrated that IGF-IR may also functionally interact with the non-canonical estrogen receptor GPER. Indeed, through the IGF-IR/PKCδ/ERK/c-fos/AP1 transduction pathway, IGF-I up-regulates GPER, which plays an important role in sustaining proliferation and migration in response to IGF-I in breast and endometrial human cancer cells [25]. In close accordance with these findings, we now show that the functional cooperation between IGF-IR and DDR1 also requires GPER, and that both DDR1 and GPER are critical to the chemotactic motility stimulated by IGF-I in mesothelioma and lung cancer cells. Notably, we now show that GPER and IGF-IR co-immunoprecipitate in lung and mesothelioma cells (Figure 2), indicating that GPER and IGF-IR also interact. Taken together all these data strongly suggest the possible formation of a ternary functional complex involving IGF-IR - DDR1 - GPER. However, further studies are needed to fully elucidate this aspect. These data may be of a particular interest as GPER expression has been associated with negative clinical features and poor survival rates in diverse types of malignancies [58-61]. In the last years, extensive studies were therefore performed in order to better characterize the role of GPER in cancer development, including the mechanisms and factors involved in its expression. For instance, we determined that EGF and IGF-I, insulin and further tumorigenic factors like hypoxia and endothelin-1 up-regulate GPER expression in diverse cancer cell contexts [25, 62-68].

Our present findings provide significant new insights on the well-established role played by the IGF axis in cancer [9-11, 14-16, 20, 23, 69-71] that involves also the interaction of IGF-IR with other RTKs and GPCRs in diverse tumor histotypes [19, 23, 72-73]. In particular, our findings might be relevant in devising new therapeutical strategies in cancers with a dysregulated IGF system. In the last decade, much effort has been made in targeting the IGF-IR in these malignancies [74]. In particular, both small-molecule IGF-IR tyrosine kinase inhibitors, and humanized monoclonal antibodies with blocking activity to the IGF-IR, have been investigated in Phase III trials of advanced non-small cell lung cancers [13]. Unfortunately, in spite of very promising preclinical studies, clinical trials have clearly indicated that only a small minority of malignancies do respond to target therapies when IGF-IR is the sole target [75], because the frequent occurrence of resistance mechanisms arising by the complex signaling network involving the IGF-IR [76].

Overall, on the basis of our data the multifaceted signaling network between IGF-IR, GPER and DDR1 could be taken into account in setting innovative combined strategies targeting these pathways in mesothelioma and lung cancers.

## MATERIALS AND METHODS

### Reagents

IGF-I, SB202190 (SB) and collagen I from rat tail were obtained from Sigma-Aldrich Inc. (Milan, Italy). PD98059 (PD) and 3-bromo-5-t-butyl-4-hydroxybenzylidenemalonitrile (AG1024) were purchased from Calbiochem (DBA, Milan, Italy). All compounds were solubilized in dimethylsulfoxide, except PD and IGF-I, which were dissolved in ethanol and in water, respectively. DDR1­IN­1 dihydrochloride (DDR-1 in) was purchased from Tocris Bioscience (Space, Milan, Italy).

### Cell cultures

IST-MES1 malignant mesothelioma cells were kindly provided by Dr. Orengo (Istituto Nazionale per la Ricerca sul Cancro, Genova, Italy). Cells were previously characterized [77] and were grown in Nutrient Mixture F-10 Ham (Ham's F-10) medium supplemented with 10% fetal bovine serum (FBS) and 100 μg/ml penicillin/streptomycin. A549 lung cancer cells were obtained by ATCC, used < 6 months after resuscitation and maintained in DMEM/F12 (Dulbecco's modified Eagle's medium) supplemented with phenol red 10% FBS and 100 μg/ml penicillin/streptomycin. All cell lines were cultured at 37°C in 5% CO_2_ and switched to medium without serum the day before immunoblots and reverse transcription-PCR experiments.

### Plasmids and luciferase assays

The GPER luciferase expression vector (promGPER) was previously described [65]. The CTGF luciferase reporter plasmid (promCTGF) (-1999/+ 36)-luc was a gift from Dr. Chaqour. EGR1-luc plasmid, containing the -600 to +12 5’-flanking sequence from the human EGR1 gene, was kindly provided by Dr. Safe (Texas A&M University). The plasmid DN/cfos, which encodes a c-fos mutant that heterodimerizes with c-fos dimerization partners but does not allow DNA biding [78], was a kind gift from Dr C Vinson (NIH, Bethesda, MD, USA). The Renilla luciferase expression vector pRL-TK (Promega, Milan, Italy) was used as internal transfection control. Cells (1x10^5^) were plated into 24-well dishes with 500 μl/well culture medium containing 10% FBS. Transfection were performed using X-treme GENE 9 DNA transfection reagent as recommended by the manufacturer (Roche Diagnostics, Milan, Italy), with a mixture containing 0.5 μg of reporter plasmid and 10 ng of pRL-TK. After 24 h, treatments were added and cells were incubated for 18 h. Luciferase activity was measured using the Dual Luciferase Kit (Promega, Milan, Italy) according to the manufacturer's recommendations. Firefly luciferase activity was normalized to the internal transfection control provided by the Renilla luciferase activity. Normalized relative light unit values obtained from cells treated with vehicle were set as 1-fold induction upon which the activity induced by treatments was calculated.

### Gene silencing experiments

Cells were plated onto 10-cm dishes and transfected by X-treme GENE 9 DNA Transfection Reagent for 24 h before treatments with a control vector, a specific shRNA sequence for each target gene. The shIGF-IR and the respective control plasmids (shRNA) were purchased from SA Bioscience Corp. (Frederick, MD, USA) and used according to the manufacturer's recommendations. The short hairpin (sh)RNA constructs to knock down the expression of GPER and the unrelated shRNA control construct have been described previously [64].

### Gene expression studies

Total RNA was extracted and cDNA was synthesized by reverse transcription as previously described [79-80]. The expression of selected genes was quantified by real-time PCR using Step One sequence detection system (Applied Biosystems, Milan, Italy). Gene-specific primers were designed using Primer Express version 2.0 software (Applied Biosystems Inc. Milan, Italy) and are as follows: GPER Fwd 5′- ACACACCTGGGTGGACACAA-3′ and Rev 5′-GGAGCCAGAAGCCACATCTG-3’; HES-1 Fwd 5′-TCAACACGACACCGGATAAA-3′ and Rev 5′-CCGCGAGCTATCTTTCTTCA-3′; NOTCH 1 Fwd 5′-AATGGCGGGAAGTGTGAAGC-3′ and Rev 5′-GCATAGTCTGCCACGCCTCT-3′; MTN-2 Fwd 5′-CTCCGAGTGGGCCAGTAAAG-3′ and Rev 5′- CTGGCTCAGATTCTGTTGGCT-3′; FBN-1 Fwd 5′-GCCGCATATCTCCTGACCTC-3′ and Rev 5′-GTCGATACACGCGGAGATGT-3′; 18S Fwd 5′- GGCGTCCCCCAACTTCTTA-3′ and Rev 5′-GGGCATCACAGACCTGTTATT-3′. Assays were performed in triplicate and the results were normalized for 18S expression and then calculated as fold induction of RNA expression.

### Western blot analysis

Cells were processed according to a previously described protocol [81] to obtain protein lysate that was electrophoresed through a reducing SDS/10% (w/v) polyacrylamide gel, electroblotted onto a nitrocellulose membrane and probed with primary antibodies against antiphosphotyrosine antibody (4G10) (Merck Millipore, Milan, Italy), IGF-IR (7G11), GPER (N-15), CTGF (L-20), phosphorylated ERK1/2 (E-4), ERK2 (C-14), NOTCH 1 (C-20), EGR1 (588), phosphorylated p-38 (D-8), p-38 (A-12), β-actin (C2), (Santa Cruz Biotechnology, DBA, Milan, Italy). Proteins were detected by horseradish peroxidase-linked secondary antibodies (DBA, Milan, Italy) and revealed using the ECL System (GE Healthcare).

### Co-immunoprecipitation

Cells were lysed using 200 μl RIPA buffer with a mixture of protease inhibitors containing 1.7mg/ml aprotinin, 1mg/ml leupeptin, 200mmol/L phenylmethylsulfonyl fluoride, 200mmol/L sodium orthovanadate, and 100mmol/L sodium fluoride. A total of 100 μg proteins were incubated for 2 h with 2 μg of the appropriate antibody (GPER, N-15; IGF-1R, 7G11) and 20 μl of protein A/G agarose immunopreciptation reagent (Santa Cruz Biotechnology, DBA, Milan, Italy). Samples were centrifuged at 13,000 rpm for 5 min at 4°C to pellet beads. After four washes in PBS, samples were resuspended in RIPA buffer with protease inhibitors and SDS sample buffer. Western Blot analysis was performed as described above.

### Migration assay

Migration assays were performed using Boyden chambers (Costar Transwell, 8 mm polycarbonate membrane, Sigma Aldrich, Milan, Italy). Cells were transfected in regular growth medium. After 8 h, cells were trypsinized and seeded in the upper chambers. Treatments were added to the medium without serum in the bottom wells where applicable, cells on the bottom side of the membrane were fixed and counted 8 hours after seeding.

### Time-lapse microscopy

Cells (1 × 10^5^) were seeded in 6-well plates and maintained in regular growth medium for 24 h. For knockdown experiments, cells were transfected for 24 h with shRNA constructs directed against IGF-IR or GPER and with an unrelated shRNA construct. Thereafter, cells were treated and transferred into a time-lapse microscopy platform, equipped with a heated stage chamber (Cytation™3 Cell Imaging Multi-Mode Reader, Biotek, Winooski, VT). Cells were maintained at routine incubation settings (37 °C, 5% CO_2_) using temperature and gas controllers. To evaluate chemotaxis the images were recorded using Cytation 3 Cell Imaging Multimode Reader and the software Gen5 (BioTek, Winooski, VT) in 10 min intervals for 8 hours. Then, the images were processed as a movie using the software Adobe Creative Cloud Premier Pro CC. Frames collected every 10 minutes are displayed at a rate of 10 frames s-^1^.

### Statistical analysis

Statistical analysis was performed using ANOVA followed by Newman-Keuls’ testing to determine differences in means. *P* < 0.05 was considered as statistically significant.

## SUPPLEMENTARY MATERIALS VIEDOS
























